# Selective Behaviour of Honeybees in Acquiring European Propolis Plant Precursors

**DOI:** 10.1007/s10886-016-0708-9

**Published:** 2016-06-13

**Authors:** Valery A. Isidorov, Sławomir Bakier, Ewa Pirożnikow, Monika Zambrzycka, Izabela Swiecicka

**Affiliations:** Forest Faculty, Białystok University of Technology, 17-200 Hajnówka, Poland; Department of Microbiology, Institute of Biology, University of Białystok, 15-950 Białystok, Poland

**Keywords:** *Apis mellifera*, Propolis, Downy birch, *Betula pubescens*, Silver birch, *Betula pendula*, Common aspen, *Populus tremula*, Black poplar, *Populus nigra*, Horse-chestnut, *Aesculus hippocastanum*, Black alder, *Alnus glutinosa*, Scots pine, *Pinus sylvestris*

## Abstract

Honey bees harvest resins from various plant species and use them in the hive as propolis. While there have been a number of studies concerning the chemical composition of this antimicrobial product, little is known about selective behavior and bee preference when different potential plant sources of resin are available. The main objective of this paper was to investigate some aspects of behavioral patterns of honeybees in the context of resin acquisition. Samples of propolis originating from temperate zones of Europe and the supposed botanical precursors of the product were analyzed. Taxonomical markers of bud resins of two white birch species, aspen, black poplar, horse-chestnut, black alder, and Scots pine were determined through GC-MS analysis. All these trees have been reported as sources of propolis, but comparisons of the chemical composition of their bud resins with the compositions of propolis samples from seven European countries have demonstrated the presence of taxonomical markers only from black poplar, aspen, and one species of birch. This suggests selective behavior during the collection of bud resins by honeybees. To examine the causes of such selectivity, the antimicrobial properties of bud resins were determined. Horse-chestnut resins had lower antimicrobial activity than the other resins which did not differ significantly.

**Electronic supplementary material:**

The online version of this article (doi:10.1007/s10886-016-0708-9) contains supplementary material, which is available to authorized users.

## Introduction

Propolis is a mixture of wax and resin exudate collected by honeybees from buds of various trees (Bankova et al. 2000). Bud resins with addition of bee salivary enzymes (Kaczmarek and Dębowski 1983) and beeswax have been used in the hive not only as a building material to seal hive walls and strengthen comb cells but also as an antimicrobial agent against a variety of pathogens (Simone-Finstrom and Spivak 2010). Apart from *Varroa destructor* mites and viruses, other pathogens include bacteria, fungi, and protozoa (Evans and Schwarz 2011; Shimanuki and Knox 2000). These pathogens are common in the honeybees’ natural environment and are brought into the hive by worker bees together with nectar, pollen, and water (Chechetkina et al. 2010). Owing to its antimicrobial properties, propolis can reduce disease at the colony level (Simone-Finstrom and Spivak 2010) and provide social immunity to the bee family (Evans and Spivak 2010). Thus, resin acquisition is a social process that is controlled by the bee colony as a whole (Nakamura and Seeley 2006).

Propolis antimicrobial activity has been attributed to flavonoid aglycones, and phenolic and hydroxycinnamic acids. The content in bud resins of different plant species varies widely (Bankova et al. 2006). It is believed that the main precursors of European and North American propolis are resins from buds of different poplar species (Bankova et al. 2000; Greenaway and Whatley 1990; Greenaway et al. 1988, 1990; Popravko 1978; Wilson et al. 2013). Other plant resins reported as precursors of propolis in the temperate zone of the Northern Hemisphere include resins of aspen and silver birch (Popravko et al. 1983, 1985), as well as pine, alder, horse-chestnut, elm, ash, oak, and beech (Crane 1990; Ghisalberti 1979; Greenaway et al. 1988; König 1985; Markham et al. 1996; Simone-Finstrom and Spivak 2010). Mixed types of propolis containing exudates of more than one plant species have been reported (Bankova et al. 2002; Isidorov et al. 2014a; Popova et al. 2005, 2013). This prompts the question as to whether honeybees show selectivity when collecting propolis precursors from available plant sources.

Bornean stingless bees have been shown to make choices by collecting resin from some plants rather than others (Leonhardt and Blüthgen 2009), and Wilson et al. (2013, 2015) found that honeybees from their apiary discriminately foraged for resin from two American poplar species, *Populus deltoides* and *P. balsamifera*, and did not collect other resins from even closely related plants. Availability, proximity, and perhaps toxicity played a role in the selection of resins (Wilson et al. 2013). However, the authors did not analyze the chemical composition of resins and propolis, and little is known about the botanical precursors of propolis in other regions, such as the boreal zone of Eurasia.

The aims of this paper were to determine the chemical composition of resin from plants assumed to be sources for propolis in Europe and to explore their benefits to bees. The chemical compositions of bud resins from seven potential plant precursors were compared with the compositions of propolis from different climatic zones of Europe, and the antimicrobial properties of the materials were measured. To our knowledge, the antimicrobial activities of bud resins from trees typically found in Europe have not been analyzed previously, with the exception of Scots pine and horse-chestnut (Wilson et al. 2013).

## Methods and Materials

### Chemicals

Pyridine, bis(trimethylsilyl)trifluoroacetamide (BSTFA) containing 1 % trimethylchlorosilane, and dimethyl sulfoxide (DMSO) were purchased from Sigma-Aldrich (Poznan, Poland). Extractions were carried out with diethyl ether (POCH SA, Gliwice, Poland).

### Propolis Samples

All samples of propolis were collected in late summer in the second half of July and August of 2014. Three propolis samples (Pr-1, Pr-2, and Pr-6) originated from the same apiary located in North Eastern Latvia (57^o^ 8.5′ N; 26^o^ 27′ E); two samples, Pr-4 and Pr-5 were collected by Russian apiarists, respectively, in the Vologda region (Pr-4) (59^o^ 58′ N; 38^o^ 31′ E) and the Udmurt Republic (58^o^ 14′ N; 52^o^ 07′ E). Single samples were supplied from Finland (Pr-3) (61^o^ 43′ N; 25^o^ 26′ E), North Eastern Poland, Bialystok region (Pr-7) (53^o^ 14′ N; 23^o^ 42′ E), Ukraine, Poltava region (Pr-8) (48^o^ 43′ N; 33^o^ 29′), Slovakia (Pr-9) (48^o^ 16′ N; 17^o^ 30′ E), and France, Rhône-Alpes Region (Pr-10) (45^o^ 07′ N; 5^o^ 20′ E). Propolis samples Pr-1, Pr-2, Pr-6, and Pr-7 were collected by the authors in August 2014. To acquire the material, a terylene net (mesh size of 1 mm) was mounted just above the hive frames with the brood. After 3 wk., the net became glued with pure propolis by the bees, and this was easily removed after cooling to −18 °C. The remaining samples (10–15 g of each) were gathered in the summers of 2013 and 2014 by apiarists from the different countries.

### Plant Material

Buds were gathered in August–September, 2014 from 20–35 yr.-old trees with up to 40–50 buds from 5–6 trees of each taxon, and were kept at -18 °C before use. Buds of downy birch (*Betula pubescens* Ehrh.) and silver birch (*B. pendula* Roth) were collected from experimental plots of the Forest Institute of the Russian Academy of Sciences, near Petrozavodsk, Russia. In order to identify birch species, the previously described method was used (Isidorov et al. 2014b). In short, the genomic DNA was extracted from parts of birch leaves, and alcohol dehydrogenase was used to study the nuclear DNA sequences. The plant material also included two accessions *Betula pubescens* (AJ535645.1) and *B. pendula* (AJ535640.1) from GenBank (NCBI). Voucher specimens were deposited with the herbarium of the Biological Department of Petrozavodsk University, Russia.

Common aspen (*Populus tremula* L.) buds were collected in August, 2014 in the neighborhood of the apiary in Tirza, Latvia from which propolis was supplied. Buds of black poplar (*P. nigra* L.) and horse-chestnut (*Aesculus hippocastanum* L.) originated from the Arboretum belonging to the Institute of Dendrology (Kórnik) of the Polish Academy of Sciences. Buds of black alder (*Alnus glutinosa* L.) and Scots pine (*Pinus sylvestris* L.) were collected in the forests around Bialystok (Poland).

### Sample Preparation and Chemical Analysis

Propolis was cooled to −18 °C and ground. An aliquot (5 g) of the powder was transferred into a flask (50 ml) and extracted by stirring with three 25-ml portions of diethyl ether for 30 min. The combined extracts were filtered through a paper filter, and the ether was removed using a rotor evaporator to obtain a brownish residue. A portion (approx. 5 mg) of the residue was dissolved in pyridine (220 μl) and BSTFA (80 μl) was added. The mixture was sealed and heated for 30 min at 60 °C to form trimethylsilyl (TMS) derivatives.

Samples (10–20 g) of buds from each of the tree species were extracted by intensive rinsing for 30 sec in diethyl ether (50 ml). The extract was filtered, and the solvent was evaporated to dryness. TMS derivatives were prepared using the technique described above.

The solutions of the TMS derivatives were analyzed by GC–MS on an HP 6890 gas chromatograph with a MSD 5973 mass selective detector (Agilent Technologies, Santa Clara, CA, USA). The GC was fitted with an HP-5MS fused silica column (30 m × 0.25 mm i.d., 0.25 μm film thickness; Agilent) with electronic pressure control and split/splitless injector. Injector temperature was 250 °C in split (1:50) mode. Carrier gas was helium (1 ml/min) in constant flow mode. The initial oven temperature was 50 °C, rising to 310 °C at 5 °C/min, and held for 15 min. The MSD transfer line temperature was 280 °C, the MS source temperature 230 °C, and the MS quadruple temperature 150 °C. Mass spectra were obtained in electron impact mode at 70 eV scanning 41–600 amu.

### Quantification and Identification

After integration of the chromatogram, the fraction of each component in the total ion current (TIC) was calculated, and the relative content was determined as percentage of the TIC. This is not a true quantification because the ion current depends on the characteristics of the compound concerned, but this approach is applicable in comparative investigations of similar-samples (Bankova et al. 2002; Melliou et al. 2007; Popova et al. 2011; Vardar-Ünlü et al. 2008). Three replicate extractions were analyzed, and the reproducibility was expressed by relative standard deviation (RSD). On average, these amounted to 2 % for main peaks (more than 10 % of TIC), 6 % for median peaks (more than 1 % of TIC), and 18 % for small peak (≤0.5 % of TIC).

Compounds were identified tentatively from their retention indices and mass spectra using NIST and home-made mass spectra libraries. The latter contains more than 1150 spectra of TMS derivatives prepared from commercial preparations of flavonoids, other phenolics, terpenoids, aliphatic acids, and alcohols.

Linear temperature programmed retention indices (*I*^*T*^) were calculated relative to the retention times of C_10_–C_40_*n*-alkanes. These were compared with NIST collection (NIST 2013) as well as our published data (Isidorov 2015; Isidorov and Szczepaniak 2009; Isidorov and Vinogorova 2003; Isidorov et al. 2009, 2014a, b, 2015a, b; Szczepaniak and Isidorov 2011; Szczepaniak et al. 2013). The identification was considered reliable if the results of the spectral library search were confirmed by the experimental *I*^*T*^ values, i.e., if their deviation from the literature values did not exceed ±5 u.i.

Cluster analysis based on Pearson correlation and complete linkage clustering was used to visualize propolis and bud exudates with common features (Statistic Software package PAST 3.0.1) (Hammer et al. 2001).

### Microorganisms, Culture Media, and Screening for Antimicrobial Activity

The diethyl ether extracts of propolis samples and bud exudates were tested against microorganisms from international collections. Bacteria were *Staphylococcus aureus* ATCC 29213 (ATCC, American Type Culture Collection), *Bacillus cereus* ATCC 10987, *Staphylococcus schleiferi* CCM 4047 (CCM, Czech Collection of Microorganisms), and *Escherichia coli* PCM 2268. The fungus *Candida albicans* PCM 2566 (PCM, Polish Collection of Microorganisms) was also tested. Microorganisms were kept at −80 °C in storage medium (1:1 LB broth and glycerol) before inoculation onto nutrient agar (bacteria) or Sabouraud agar (fungi) and incubation overnight at 37 °C. Microbiological media were supplied by Oxoid Ltd., Basingstoke, England.

The antimicrobial activity of the extracts was assessed by determining the minimal inhibitory concentration (MIC) in accordance with the CLSI (Clinical and Laboratory Standard Institute 2011) protocols. Dry extracts were dissolved in DMSO at a concentration of 200 mg/ml, filtered through a Rotilabo-Spritzenfilter filter (0.22 μm pore size; Carl Roth GmbH and Co, Karlsruhe, Germany), and serially diluted in Mueller-Hinton broth (5–0.01 mg/mL). Aliquots (100 μl) were placed into a U-shaped 96-well microtriter plate.

Bacteria were cultured overnight in Mueller-Hinton broth at 37 °C with shaking (200 rpm). Cultures were suspended to final optical density of 0.2–0.3 at 600 nm wavelength measured with a V-670 spectrophotometer (Jasco, Japan). For the assay, 100 μl of the bacterial suspensions were added to each well in the microtriter plate containing diluted exudate or propolis extracts and incubated overnight at 37 °C. In order to obtain comparable data, all bacteria were treated under the same conditions. The MIC values were determined as the lowest concentration of the extracts in the wells with no bacterial growth observed visually. All tests were carried out in quadruplicate. As a positive control, microorganisms cultured in Mueller-Hinton broth without extracts were applied. Mueller-Hinton broth supplemented with 10 % DSMO was used as solvent control, while Mueller-Hinton broth with 10 % DMSO and extracts was used as the exudate and propolis extract control. The MIC value for *C. albicans* was assessed as above, but with Sabouraud broth instead of Mueller-Hinton broth.

## Results

### Chemical Compositions of Resin and Propolis Samples

The chemical compositions of seven potential precursors of propolis in Europe were investigated. Three other tree species, i.e., ash (*Fraxinus excelsior*), oak (*Quercus robur*), and beech (*Fagus sylvatica*) previously reported to be sources of propolis (see Introduction) were excluded because observations failed to reveal the presence of resins on the buds of these trees at any stage of their development, or from bark or other plant structures.

Table 1 shows the compositions of the resins (Ex-1–Ex-7) and the propolis samples (Pr-1–Pr-10) from seven European countries. In total, 353 compounds were detected in different samples of propolis and bud resin, and Table 1 shows the structural groups of compounds and the most abundant compounds in these groups. Realtive compositions for individual compounds and their analytical parameters (*I*^*T*^ values and molecular ions, M^+^) are presented as Supplementary Information.

**Table 1:** Relative average composition (%) of buds resins and ether extracts of propolis^a^

Compound	Bud exudate							Propolis extract									
	Downy birch, Ex-1	Silver birch, Ex-2	Aspen, Ex-3	Black poplar, Ex-4	Horse-chestnut, Ex-5	Black alder, Ex-6	Scots pine, Ex-7	Latvian, Pr-1	Latvian, Pr-2	Finnish, Pr-3	Russian,Pr-4	Russian, Pr-5	Latvian, Pr-6	Polish, Pr-7	Ukrainian, Pr-8	Slovak, Pr-9	French, Pr-10
Cinnamic acids	2.1	trace*	5.7	19.0	-	-	-	9.0	6.7	13.2	3.0	10.3	8.7	3.4	14.1	17.2	14.8
*p*-coumaric acid	2.1	trace	1.5	3.0	-	-	-	4.3	3.0	6.2	2.2	5.5	4.5	2.6	0.7	1.2	9.8
ferulic acid	trace	-	2.2	1.1	-	-	-	3.7	3.1	5.7	0.8	4.3	3.1	0.4	10.8	11.3	1.1
caffeic acid	trace	-	1.8	5.3	-	-	-	0.6	0.4	1.0	0.03	0.3	0.8	0.1	1.5	1.8	1.7
Cinnamic acid esters	-	-	10.0	24.6	-	-	-	23.4	18.0	3.9	1.6	5.7	22.6	6.8	13.2	11.4	20.3
prenyl cinnamates	-	-	-	8.5	-	-	-	-	-	-	-	-	0.8	0.7	6.2	3.2	5.8
benzyl cinnamates	-	-	2.2	7.8	-	-	-	11.0	5.9	3.2	1.2	5.4	8.0	1.4	3.3	3.9	6.1
coniferyl cinnamates	-	-	7.8	-	-	-	-	8.3	4.5	0.7	0.3	0.3	8.3	0.4	-	-	-
Phenylpropenoid glycerides	-	-	64.2	-	-		-	33.5	31.8	57.3	1.6	4.7	11.4	1.6	-	-	1.5
1,3-di-*p*-coumaroyl glycerol	-	-	2.8	-	-	-	-	1.1	2.9	10.1	0.4	0.5	0.7	0.2	-	-	0.2
2-acetyl-1,3-di-*p*-coumaroyl glycerol	-	-	10.5	-	-	-	-	5.4	5.9	14.1	0.9	2.8	4.0	1.1	-	-	1.2
2-acetyl-1-*p*-coumaroyl-3-feruloyl glycerol	-	-	13.8	-	-	-	-	2.0	3.7	6.8	0.4	0.5	2.2	0.2	-	-	0.1
2-acetyl-1,3-diferuloyl glycerol	-	-	6.5	-	-	-	-	1.7	1.6	4.5	trace	0.4	0.8	0.2	-	-	0.2
Phenylpropenoid sesquiterpenols	9.7	-	-	-	-	-	-	-	-	1.2	6.9	8.9	-	5.1	-	-	-
6-Hydroxy-β-caryophyllene *p*-coumarate	4.8	-	-	-	-	-	-	-	-	0.1	2.1	3.2	-	1.4	-	-	-
14-Hydroxy-β-caryophyllene *p*-coumarate	3.72	-	-	-	-	-	-	-	-	0.5	1.6	1.1	-	0.7	-	-	-
14-Hydroxy-β-caryophyllene ferulate	0.88	-	-	-	**-**	-	-	-	-	0.6	0.5	0.4	-	0.1	-	-	-
Total phenylpropenoids	11.8	-	73.6	43.7	-	-	-	55.8	56.5	74.4	13.2	29.6	42.6	17.0	27.3	28.5	36.7
Flavonoids	49.8	0.9	4.2	38.0	13.0	13.3	2.5	0.5	0.6	1.0	45.4	30.9	10.8	50.1	46.6	59.3	40.2
apigenin	0.1	-	0.1	0.2	-	-	-	0.2	0.3	-	-	0.4	0.2	0.6	0.1	0.3	0.2
pinobanksin	-	-	-	1.6	-	-	-	-	-	-	-	-	0.2	0.4	2.8	4.3	2.8
pinobanksin 3-acetate	-	-	-	8.2	-	-	-	-	-	-	-	-	2.0	0.6	12.8	9.3	8.5
galangin	-	-	-	5.2	-		-	-	-	-	-	-	2.0	0.5	6.5	4.5	6.8
chrysin	-	-	-	4.7	-	-	-	-	-	-	-	-	1.1	-	6.6	6.8	5.6
pinocembrin	-	-	-	5.2	-		-	-	-	0.05	-	-	0.4	1.3	9.5	7.6	11.0
sakuranetin	18.6	-	1.8	trace	-	-	-	0.1	0.2	trace	12.8	9.7	0.1	10.8	0.4	0.1	0.2
homoeriodictyol	4.9	-	-	-	-	-	-	-	0.2	-	3.8	2.7	-	3.5	-	-	-
pectolinaringenin	7.5		-	-	-	-	-	-	-	-	5.9	2.7	-	5.3	-	-	-
catechine	trace	0.3	-	-	2.2	1.3	1.9	-	-	-	-	-	-	-	-	-	-
Sesquiterpenoids	28.8	0.6	0.02	8.7	-	1.6	9.3	0.1	0.1	-	8.5	13.9	0.1	13.8	0.1	0.6	0.01
birkenal & β-betulenal	0.7	-	-	-	–	-	-	-	-	-	0.2	0.4	-	0.3	-	-	-
6-hydroxy-β-caryophyllene	6.0	-	-	-	–	-	-	-	-	-	0.33	2.6	-	3.2	-	-	-
14-hydroxy-β-caryophyllene	2.9	-	-	-	-	-	-	-	-	-	0.2	1.2	-	1.8	-	-	-
Triterpenoids & sterols	1.8	81.6	4.7	2.6	43.4	17.3	2.0	3.4	5.2	6.4	0.4	1.3	1.8	0.4	4.2	4.5	0.6
dammaradien-3-one	-	4.7	-	-	-	1.4	-	-	-	-	-	-	-	-	-	-	-
dipterocarpol	trace	33.0	-	-	-	1.2	-	-	-	-	-	-	-	-	-	-	-
Diterpenoids	-	-	-	-	-	-	78.4	-	-	-	-	-	-	-	-	-	-
3-Hydroxy C_14_-C_22_ acids	-	-	-	-	20.1	0.1	-	-	-	-	-	-	-	-	-	-	-
Glyceride of C_12_-C_22_ acids	-	-	-	-	-	46.3	-	-	-	-	-	-	-	-	-	-	-
Benzoic acid & other aromatics	-	0.2	4.2	3.7	0.1	0.6	0.4	15.7	7.4	4.8	1.2	9.7	17.9	1.0	0.3	0.1	1.0
Aliphatic C_12_-C_30_ acids	3.6	2.6	0.5	trace	29.5	2.6	1.6	7.8	18.7	0.4	14.4	3.9	14.2	8.6	2.4	0.1	15.7
Aliphatic alcohol	0.2	0.5	1.5	0.2	0.2	3.0	0.5	0.2	1.4	1.6	0.4	0.03	1.3	0.4	0.7	-	0.2
Alkanes & alkenes	1.4	1.4	0.7	0.9	0.04	3.6	-	6.4	5.7	1.3	9.2	3.8	7.7	6.9	5.2	-	4.4
Non identified	2.6	12.2	10.5	2.3	13.7	11.9	7.8	10.1	4.9	8.8	7.0	5.5	3.6	1.9	13.3	6.8	2.6

^a^trace – below 0.01 % of TIC

### Taxonomic Markers in Resins

**Fig. 1:**
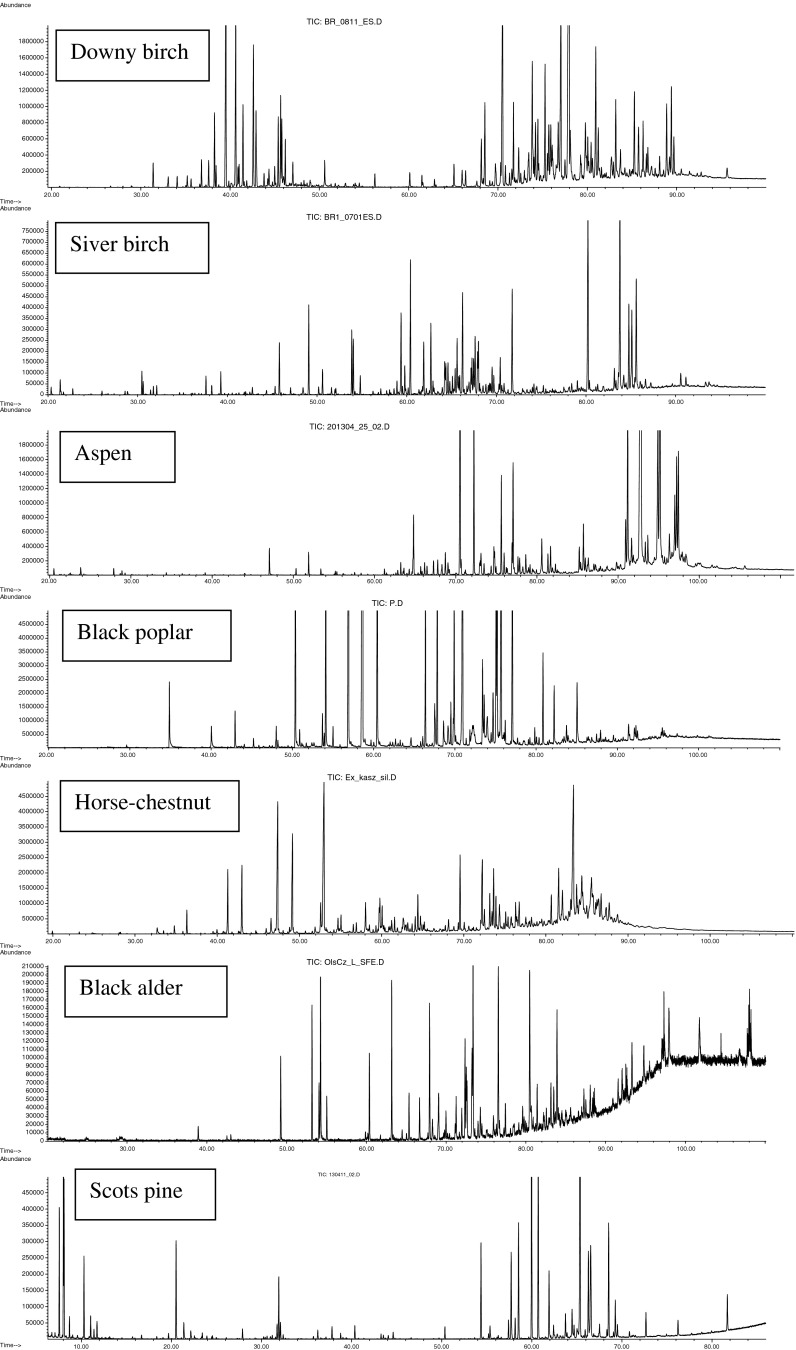
GC-MS chromatograms of ether extracts from buds of potential botanical precursors of propolis. The *x*-axis gives time (min), and the *y*-axis gives abundance of chromatographic signals

GC-MS traces from analyses of the resins are shown in Fig. 1. For the two European species of white birch, the distinguishing feature of downy birch (*B. pubescens*) bud resin (Ex-1) was a high level of flavanone-type flavonoids such as sakuranetin, homoeriodictyol, and pectolinaringenin (31.0 % of TIC) along with characteristic phenylpropenoids, esters of hydroxycinnamic (*p*-coumaric, ferulic, and caffeic) acids with sesquiterpene alcohols of caryophyllane and humulane series, as well as *nor*-sesquiterpenoids, birkenal and birkenol. In contrast, the resin from silver birch (*B. pendula* Roth. ≡ *B. verrucosa* Her.) buds (Ex-2) contained only relatively low levels of phenolics, being mostly composed of triterpenoids (up to 80 % of TIC), and two compounds, dammaradien-3-one and dipterocarpol, can be used as taxonomical markers of *B. pendula*.

The common or quaking aspen (*P. tremula*) (Ex-3) is a species native to cold regions of Europe and Asia. It is distinguished from other trees by the presence of other groups of phenylpropenoids in its buds, such as esters of hydroxycinnamic acids and glycerol (up to 64 %). Another group of phenylpropenoids specific to aspen is esters of coniferyl alcohol and hydroxycinnamic acids, coniferyl *p*-coumarate, coniferyl ferulate, and coniferyl caffeate (8 % of TIC). However, levels of flavonoid aglycones in these extracts were below 5 % of TIC.

The resin of black poplar (*P. nigra*) (Ex-4) contained hydroxycinnamic acids as well as their esters and flavonoids. The latter included flavones such as apigenin and chrysin, flavanone pinocembrin, and flavonols such as galangin, pinobanksin, and pinobanksin 3-acetate. The distinguishing feature of this resin was that it contained specific phenylpropenoids, prenylated *p*-coumarate, ferulate, and caffeate.

Buds of horse-chestnut (*Aesculus hippocastanum*) (Ex-5) have abundant resin but have not been investigated previously. This resin contained relatively small amounts of flavonoids (13.0 %) but considerable amounts of triterpenoids (43.4 %). However, the distinguishing feature was a relatively high level of C_14_–C_22_ aliphatic 3-hydroxyacids (20.1 %).

Black alder (*Alnus glutinosa*) buds (Ex-6), also are rich in viscous resin and possessed relatively low levels of flavonoids (13.3 %). The main group of resin constituents was monoglycerides of aliphatic C_12_–C_22_ acids (46.3 %). These substances give the exudate its sticky properties.

Scots pine (*Pinus sylvestris*) bud resins (Ex-7), as well as the secretions from damaged tissues of this coniferous plant, rapidly crystallize. Their chemical composition is characterized by high levels of diterpenoids (78.4 %), mainly diterpene acids such as neoabietic (11.9 %), pinifolic (10.3 %), dehydroabietic (3.6 %), and isopimara-8,15-dienoic acid (3.3 %).

### Comparison of Compositions of Resins and Propolis

Comparison of the chemical composition of resins and samples of propolis indicated that the main botanical precursors of Latvian (Pr-1 and Pr-2) and Finnish propolis (Pr-3) were the exudate originating from aspen buds, which contained only phenylpropenoid glycerides. Samples Pr-1 and Pr-2 from Latvia were almost pure “aspen-type” propolis, whereas the third sample of Latvian propolis Pr-6 contained a small percentage of components typical of black poplar such as prenyl cinnamates and characteristic flavonoids (chrysin, pinocembrin, pinobanksin-3-acetate, and galangin). All three samples, i.e., Pr-1, Pr-2, and Pr-6 were collected at the same time (between 25 July and 15 August 2014) by honeybees of the same race (*Apis mellifera carnica*) living in neighboring hives, and aspen and black poplar were the closest tree species.

The Finnish propolis Pr-3 contained small amounts (1.2 %) of phenylpropenoid sesquiterpenols, taxonomic markers of downy birch. Although Russian propolis samples Pr-4 and Pr-5 clearly resembled downy birch bud resin, they also contained phenylpropenoid glycerides typical of aspen buds.

The Polish propolis Pr-7 contained compounds from three plant precursors, downy birch, aspen, and black poplar. This sample was collected from the hives in an apiary located on the outskirts of a mixed forest formed by Scots pine, aspen and silver birch with a few black poplars in the vicinity of the apiary. It also is important that there was no downy birch in the neighborhood of the apiary. This birch species was found relatively far away (about 0.5 km) but within common foraging range (2–3 km).

Samples of propolis from the almost woodless region of the Ukraine (Poltava region) (Pr-8) and from the Slovak Republic (Pr-9) contained only compounds characteristic of poplar. French propolis (Pr-10) from the Rhône-Alpes region (foot of the French Alps) also showed predominantly poplar resin compounds with some phenylpropenoid glycerides, which are taxonomic markers of aspen buds.

**Fig. 2:**
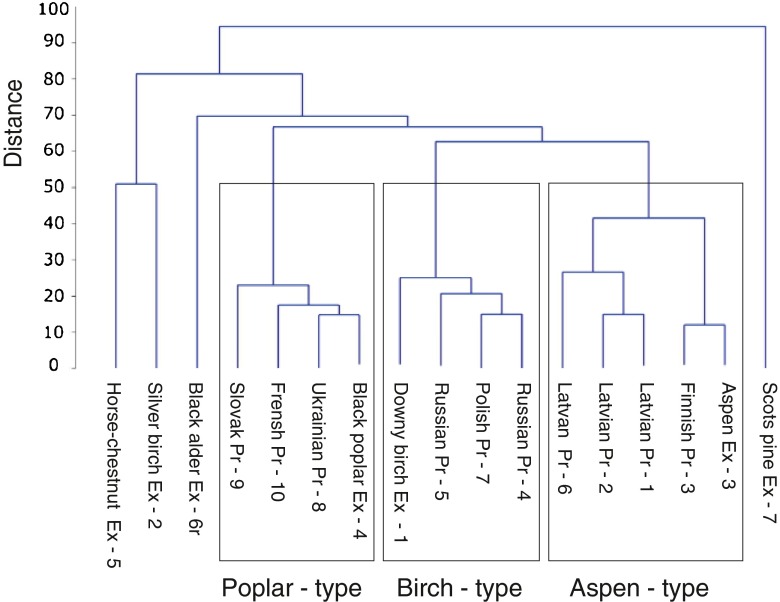
Dendrogram for the chemical similarity of tree species resins and samples of propolis

None of the propolis samples in this paper or in our earlier work (Isidorov et al. 2014b) contained any taxonomic markers of silver birch (*B. pendula*), horse-chestnut, black alder, or Scotch pine in spite of the fact that the buds of all of these trees are rich in resin. This is shown in the dendrogram in Fig. 2, which was created by making use of the concentration of specific compounds marked by asterisks in Table 1S of the Supplementary Material. The samples of propolis are divided into three separate groups associated with one of the three major botanical precursors, downy birch (Ex-1: Pr-4, Pr-5, and Pr-7), black poplar (Ex-4: Pr-8–Pr-10) or aspen (Ex-3: Pr-1–Pr-3, and Pr-6) but not silver birch (Ex-2), horse-chestnut (Ex-5), black alder (Ex-6), or Scots pine (Ex-7).

### Antimicrobial Activity

As shown in Table 2, both propolis and bud resins demonstrated higher activity (lower MIC) against Gram-positive bacteria and *C. albicans* fungus. The inhibitory action on Gram-negative *P. aeruginosa* was less pronounced; however, the extracts did not inhibit *E. coli.* Horse-chestnut resins had lower antimicrobial activity than other resins. However, the antimicrobial activities of black alder and Scotch pine resins differed only marginally from the antimicrobial activities of birch, aspen and poplar resins.

**Table 2:** Antibacterial activity of bud resins and selected propolis extracts

	(Minimal Inhibitory Concentration MIC (μg/ml)												
Microorganism	Bud resin							Propolis extract					
	Downy birch (Ex-1)	Silver birch (Ex-2)	Aspen (Ex-3)	Black poplar (Ex-4)	Horse-chestnut (Ex-5)	Black alder (Ex-6)	Scots pine (Ex-7)	Latvian (Pr-1)	Latvian (Pr-2)	Finnish (Pr-3)	Russian (Pr-4)	Russian (Pr-5)	Latvian (Pr-6)
Gram-positive bacteria													
*Staphylococcus aureus*	39	78	39	78	156	20	20	62	16	62	16	16	16
*Staphylococcus schleiferi*	39	39	39	39	312	78	156	31	31	31	31	31	31
*Staphylococcus saprophyticus*	39	39	39	39	312	78	78	31	16	31	31	16	31
*Bacillus thuringiensis*	78	78	39	78	156	78	39	31	31	62	31	16	31
*Bacillus cereus*	78	156	39	78	312	78	39	62	62	62	62	31	62
Gram-negative bacteria													
*Pseudomonas aeruginosa*	250	500	250	500	250	250	250	250	250	500	500	500	500
*Escherichia coli*	>5000	>5000	>5000	>5000	>5000	>5000	>5000	2500	5000	5000	5000	5000	2500
Fungus													
*Candida albicans*	32	64	-	32	64	32	39	156	39	39	78	312	78

## Discussion

The buds of two birch species, as well as aspen, black poplar, horse-chestnut, black alder, and Scotch pine typically are coated in sticky resins, and these trees are widespread in the temperate zone of the Northern Hemisphere. However, on the northern border of the temperate zone, black poplar is rare and occurs mainly as an ornamental plant. In the southern part of that zone, characterized by a warmer and drier climate, birch, aspen, black alder, and Scotch pine can be found only in mountain forests (Flora 1981). These peculiarities influence the chemical composition of propolis from different parts of the zone.

The first conclusion that can be drawn from the data here is that when a variety of sources of resin are available to honeybee colonies, the composition of the propolis rarely corresponds exclusively to only one plant precursor. A similar conclusion was reached previously based on analyses of propolis from 11 countries in Europe and Asia (Isidorov et al. 2014b). Resin diversity may be more beneficial for bees in respect to protection against multiple pathogens because of synergistic action of components originating from various plant materials (Drescher et al. 2014).

The second conclusion concerns the selective activity of honeybees while collecting resins. None of the propolis discussed in this paper or examined in our earlier work (Isidorov et al. 2014b) contained any taxonomic markers of silver birch (*B. pendula*), horse-chestnut, black alder, or Scotch pine despite the fact that the buds of all these trees are rich in resin. Wilson et al. (2013) also failed to find traces of the latter two plants in resin samples collected from honeybees’ corbiculae, despite the fact that the plants occurred in the study area. Thus, honeybees seem to gather resin from some types of trees while ignoring others (even closely related), such as the two different species of white birches.

There are some discrepancies between our data and those reported by other authors. Russian chemists (Popravko et al. 1985) as well as the authors of a recent publication (Popova et al. 2013) reported the resin of silver birch (*B. pendula*) to be the plant precursor of propolis in Russia. Other authors (Kononenko et al. 1975; Popravko et al. 1979) have reported high levels of flavonoid aglycones in silver birch buds, which were not detected in the present work. However, they constitute the main group of secondary metabolites of downy birch (Isidorov et al. 2014b). These reports do not specify how the birch species were identified, and it is possible they were misidentified given the taxonomic problems in the *Betula* L. genus (Migalina et al. 2010).

Another discrepancy concerns diterpene acids, which form the main group of compounds in *P. sylvestris* bud exudate but are not found in temperate zone propolis. However, these compounds have been identified in propolis from Greece, the Aegean Sea Islands, and Cyprus (Kalogeropoulos et al. 2009; Melliou and Chinou 2004; Popova et al. 2009). Their precursors in this region are conifer plants of Cupressaceae and Pinaceae families. The latter are presented by subtropical species such as *Pinus pinea*, *P. nigra*, and *P. halepensis*, as well as Turkish pine *P. brutia*, characteristic of the East Aegean Sea Islands and Cyprus.

Two hypotheses may be considered to explain the selective behavior of honeybees during resin acquisition. The first refers to the antimicrobial properties of plant precursors, as the main function of propolis in the bee family is protection against pathogenic microorganisms (Evans and Spivak 2010; Simone-Finstrom and Spivak 2010). The other hypothesis concerns the possible presence of deterrent or even toxic compounds (Wilson et al. 2015) in bud exudates, which should discourage honeybees from collecting these specific bud resins.

In order to examine the first hypothesis, the antimicrobial activity of bud resins was compared with the activity of ether extracts in six selected propolis samples, by using microbes found in nectar- and pollen-giving plants (Chechetkina et al. 2010). Gram-positive bacteria were represented by two strains of *Staphylococcus* sp. and *Bacillus cereus*. Gram-negative bacteria *Escherichia coli* and *Pseudomonas aeruginosa* are highly pathogenic to insects. It is known that *P. aeruginosa* is the etiological agent of septicemia (Shimanuki and Knox 2000), which occurs in honey bees (Bailey and Ball 1991).

Despite the differences in the chemical composition of the propolis samples, all showed similar activity in relation to the microorganisms examined. The resins discriminated against by honeybees such as silver birch, black alder, and Scots pine demonstrated rather high antibiotic activity. Thus, the antimicrobial assay fails to provide definite evidence for why honeybees decline to gather specific material. This conclusion corresponds to data from Wilson et al. (2013), who investigated the action of different plant resins on *Paenibacillus larvae* to explore the possibility of antimicrobial activity as a criterion for resin preference. The results showed that honeybees collect poplar *Populus deltoides* resins characterized by moderate antimicrobial activity (MIC 0.175 mg/ml) but not the much more active (MIC 0.05–0.06 mg/ml) resins from coniferous trees (*Picea glauca*, *Pinus banksiana*, and *Pinus ponderosa*).

As for the other hypothesis, there is presently no candidate to be considered as a deterrent or repellent compound. Therefore, the suggestion of a repulsive, deterrent or toxic action of certain resin components on honeybees (Wilson et al. 2013) requires further research.

## Electronic Supplementary Material

ESM 1 (DOC 1047 kb)
