# Supplementary material for: Selective Behaviour of Honeybees in Acquiring European Propolis Plant Precursors
Source: J Chem Ecol. 2016 Jun 13;42:475–85. doi: 10.1007/s10886-016-0708-9 (PMC4947481; doi:10.1007/s10886-016-0708-9)
Supplement: Supplementary file 1 — (DOC 1047 kb) [file 10886_2016_708_MOESM1_ESM.doc]

**Selective Behavior of European Honeybees in Acquiring Propolis Plant Precursors**

**Valery A. Isidorov^1^*, Sławomir Bakier^1^, Ewa Pirożnikow^1^, Monika Zambrzycka^2^, Izabela Swiecicka^2^**

*^1^Forest Faculty, Białystok University of Technology, 17-200 Hajnówka, Poland*

*2Institute of Biology, Department of Microbiology, University of Białystok, 15-950 Białystok, Poland*

**Supplementary Data**

Solutions of the TMS derivatives were analyzed by GC–MS on an HP 6890 gas chromatograph with a MSD 5973 mass selective detector (Agilent Technologies, Santa Clara, CA, USA). The GC was fitted with a HP-5MS fused silica column (30 m × 0.25 mm i.d., 0.25 μm film thickness; Agilent) with electronic pressure control and split/splitless injector. Injector temperature was 250 ^o^C in split (1:50) mode. Carrier gas was helium (1 ml/min) in constant flow mode. The initial oven temperature was 50°C, rising to 310°C at 5°C/min, and held for 15 min. The MSD transfer line temperature was 280°C, the MS source temperature 230°C and the MS quad temperature 150°C. Mass spectra were obtained in electron impact mode at 70 eV scanning 41–600 atomic mass units.

**Table 1S.** Chemical composition (% of TIC) of extracts from tree buds and selected propolises (Bud exudate: Ex-1 – downy birch (*B. pubescens*); Ex-2 – silver birch (*Betula pendula*); Ex-3 – aspen

(*P. tremula*); Ex-4 – black poplar (*P. nigra*); Ex-6 – black alder (*Alnus glutinosa*); Ex-7 – pine (*Pinus sylvestris;* Propolis: Latvian (Pr-1, Pr-2), Russian (Pr-5), Polish (Pr-7), French (Pr-10); . asterisk refers to those compounds which were used in the dendrogram (Fig 2) creation.

| Compound, TMS | *I^T^* | M+ | Bud exudate | | | | | | Propolis | | | | |
| --- | --- | --- | --- | --- | --- | --- | --- | --- | --- | --- | --- | --- | --- |
|  |  |  | Ex-1 | Ex-2 | Ex-3 | Ex-4 | Ex-6 | Ex-7 | Pr-1 | Pr-2 | Pr-5 | Pr-7 | Pr-10 |
| Lactic acid | 1069 | - | 0.02 | - | - | - | 0.05 | - | - | - | - | - | - |
| Benzyl alcohol | 1157 | 180 | - | - | 0.05 | - | - | - | 0.01 | 0.02 | - | - | - |
| Malonic acid | 1216 | - | - | 0.10 | - | - | - | - | - | trace | - | - | - |
| 2-Phenyl ethanol | 1228 | - | - | trace | - | 0.09 | - | - | - | - | - | - | - |
| Benzoic acid | 1245 | 194 | - | trace | 0.74 | 2.55 | - | 0.42 | 3.59 | 6.73 | 4.3 | 0.03 | 0.21 |
| Phosphoric acid | 1289 | 314 | 0.01 | trace | - | - | 0.01 | - | - | - | - | - | - |
| Glycerol | 1293 | - | 0.01 | - | 0.03 | 0.11 | 0.12 | - | 0.05 | 0.03 | 0.12 | 0.07 | 0.05 |
| Succinic acid | 1323 | 262 | 0.01 | - | - | - | - | - | - | - | - | trace | 0.06 |
| Nonanoic acid | 1366 | - | - | - | - | - | 0.08 | - | - | - | - | - | - |
| 4-Hydroxybenzaldehyde | 1367 | 194 | - | - | - | - | - | - | 0.03 | 0.05 | trace | 0.01 | - |
| α-Copaene | 1376 | 204 | - | 0.05 | - | - | - | - | - | - | - | - | - |
| β-Bourbonene | 1384 | 204 | - | - | - | - | 0.03 | - | - | - | - | - | - |
| Hydroquinone | 1405 | 254 | - | - | - | - | - | - | 0.03 | 0.02 | trace | - | 0.06 |
| β-Caryophyllene | 1417 | 204 | 0.13 | trace | - | - | 0.01 | 0.22 | - | 0.03 | - | 0.02 | - |
| Hydrocinnamic acid | 1418 | 222 | - | - | - | 0.23 | - | - | - | - | - | - | 0.12 |
| Cinnamyl alcohol | 1429 | 206 | - | - | - | trace | - | - | - | - | - | - | - |
| Birkenal* | 1438 | 206 | 0.54 | - | - | - | - | - | 0.04 | 0.04 | 0.10 | 0.12 | - |
| β-Guaiene | 1444 | 204 | - | trace | - | - | - | - | - | - | - | - | - |
| Eugenol | 1452 | 236 | trace | - | - | - | - | - | - | - | - | - | - |
| α-Humulene | 1455 | 204 | 0.03 | trace | - | - | - | - | - | - | - | 0.01 | - |
| 9-*epi*-Caryophyllene | 1462 | 204 | - | 0.02 | - | - | - | - | - | - | - | - | - |
| 4-Hydroxyacetophenone | 1476 | 208 | - | - | - | - | - | - | - | - | - | 0.01 | 0.05 |
| Germacrene D | 1480 | 204 | - | 0.13 | - | - | 1.08 | 0.38 | - | - | - | - | - |
| γ-Himachalene | 1484 | 204 | - | - | - | - | - | 0.14 | - | - | - | - | - |
| Prenyl benzoate | 1492 | - | - | - | - | - | - |  | - | - | - | 0.01 | - |
| α-Muurolene | 1499 | 204 | - | - | - | - | - | 0.41 | - | - | - | - | - |
| γ-Cadinene | 1513 | 204 | - | - | - | - | - | 0.50 | - | - | - | - | - |
| 4-Methoxybenzoic (anisic) acid | 1521 | 224 | 0.03 | 0.07 | - | - | - | - | - | - | - | 0.01 | - |
| δ-Cadinene | 1522 | 204 | - | - | - | - | - | 0.86 | - | - | - | - | - |
| Vanillin | 1533 | 224 | trace | - | 0.08 | trace | - | - | 0.59 | 0.40 | 1.53 | 0.01 | - |
| des-4-Methylcaryophyll-8(14)-en-5-one | 1536 | 206 | 0.04 | - | - | - | - | - | - | - | - | - | - |
| Cinnamic acid | 1546 | 220 | trace | - | 0.03 | 0.21 | - | - | 0.13 | 0.11 | 0.12 | 0.01 | 1.04 |
| Birkenol | 1566 | - | 0.16 | - | - | - | - | - | - | - | trace | 0.02 | - |
| Caryophylla-4(12),8(13)-dien-5-one | 1674 | 220 | 0.05 | - | - | - | -- | - | - | - | - | - | - |
| Caryophyllene oxide | 1581 | 220 | 0.32 | trace | - | - | - | - | trace | 0.08 | - | 0.12 | - |
| 4-Hydroxyphenylethanol | 1580 | 282 | - | 0.02 | - | - | - | - |  |  | - | - | - |
| Humulene epoxide II | 1603 | 220 | 0.08 | 0.01 | - | - | - | - | trace | 0.03 | - | 0.02 | - |
| Protocatecaldehyde | 1618 | 282 | - | - | 0.04 | 0.14 | - | - | 0.10 | 0.14 | trace | - | - |
| 4-Hydroxybenzoic acid | 1629 | 282 |  | trace | 0.05 | 0.50 | - | - | 0.20 | 0.52 | 0.28 | - | - |
| Cubebol | 1630 | 294 | - | - | - | - | - | 0.26 | - | - | -- | - | - |
| Caryophylladien-5a-ol | 1636 | 292 | 0.59 | - | - | - | - | - | - | - | - | 0.27 | - |
| Caryophylladien-5β-ol | 1640 | 292 | 0.11 | - | - | - | - | - | - | - | - | 0.06 | - |
| C_15_H_26_O (161,204,159) | 1641 | 294 | - | - | - | - | - | 0.11 | - | - | - | - | - |
| β-Betulenal | 1647 | 218 | 0.06 | - | - | - | - | - | - | - | - | - | - |
| Dodecanoic acid | 1658 | - | - | trace | 0.05 |  | trace | - | 0.11 | 0.09 | trace | 0.01 | - |
| C_15_H_24_O-TMS (73,161,277) | 1665 | - | 0.04 | - | - | - | - | - | - | - | - | 0.07 | - |
| C_15_H_26_O-TMS (251,73,75) | 1668 | 294 | - | - | - | 0.10 | - | - | - | - | - | - | - |
| C_15_H_24_O-TMS (73,75,156) | 1675 | 292 | 0.05 | - | - | - | 0.25 | - | - | 0.32 | - | - | - |
| Germacrene D-4-ol | 1673 | 294 | - | - | - | - | 0.03 | 4.96 | - | - | - | - | - |
| C_15_H_26_O-TMS (73,93,131,105) | 1680 | 294 | - | - | - | - | 0.17 | 0.54 | - | - | - | - | - |
| 6-Hydroxy-β-caryophyllene* | 1682 | 292 | 6.01 | - | - | - | - | - | - | - | 0.33 | 3.08 | - |
| Guaiol | 1686 | 294 | - | - | - | 0.36 | - | - | - | - | - |  | - |
| C_15_H_24_O-TMS (73,208,292) | 1692 | 292 | 0.13 | - | - | - | - | - | - | 0.32 | trace | 0.05 | - |
| τ-Cadinol | 1703 | 294 | - | 0.02 | - | - | - | 0.25 | - | - | - | - | - |
| 14-Hydroxy-β-caryophyllene* | 1706 | 292 | 2.89 | - | - | - | - | - | - | - | 0.21 | 0.93 | - |
| Caryophylla-3,8(13)-dien-5α-ol acetate | 1738 | 262 | 0.09 | - | - | - | - | - | - | - | - | - | -- |
| Betuligenol | 1722 | 310 | - |  |  |  | - | 0.10 |  |  | - | - | - |
| 14-Hydroxyisocaryophyllene | 1725 | 292 | 0.75 | - | - | - | - | - | - | - | 0.08 | 0.75 | - |
| Caryophylla-4(12),8(13)-dien-5α-ol acetate | 1738 | - | 0.03 | - | - | - | - | - | - | 0.03 | - | - | - |
| γ-Eudesmol-TMS | 1743 | 294 | - | - | - | 1.80 | - | - | - | - | - | - | - |
| α-Cadinol | 1747 | 294 | - | 0.09 | - | - | - | - | - | - | - | - | - |
| β-Eudesmol | 1748 | 294 | - | - | - | 1.69 | - | - | - | - | - | - | - |
| α-Eudesmol-TMS | 1750 | 294 | - | 0.05 | - | 0.38 | - | - | - | - | - | - | - |
| 6-Hydroxy-β-caryophyllene acetate | 1756 | 262 | 0.79 | - | - | - | - | - | - | - | 0.05 | 0.22 | - |
| Benzyl benzoate | 1759 | 212 | - | - | 0.43 | - | - | - | 0.68 | 0.73 | 0.34 | 0.24 | - |
| 3- Hydroxy-β-caryophyllene acetate | 1761 | 262 | 0.29 | - | - | - | - | - | - | - | trace | - | -- |
| 14-Hydroxy-α-humulene acetate | 1773 | 262 | 0.64 | - | - | - | - | - | - | - | trace | 0.22 | - |
| Vanillic acid | 1771 | 312 | 0.10 | - | - | - | - | - | 0.02 | 0.03 | trace | - | - |
| α-Eudesmol acetate | 1783 | 264 | - | - | - | - | - | - | - | 0.15 | - | - | - |
| 14-Hydroxy-β-caryophyllene acetate* | 1791 | 262 | 5.48 | - | - | - | - | - | - | - | 0.47 | 1.17 | - |
| (Z)-*p*-Coumaric acid | 1795 | 308 | - | - | - | - | - | - | 0.15 | 0.23 | 0.46 | 0.29 | 0.04 |
| C15H24O2-mono-TMS (132,73)* | 1799 | 308 | 1.08 |  |  |  | - |  | 0.05 |  | - | 0.12 | - |
| 4-Hydroxycinnamyl alcohol | 1803 | 294 | - | - | - | - | - | - | 0.06 | 0.11 | - | - | - |
| C_15_H_24_O_2_-mono-TMS | 1804 | 308 | 0.48 | - | - | - | - | - | - | - | - | 0.42 | - |
| Azelaic acid | 1808 | - | - | 0.02 | - | - | - | - | - | - | - | - | - |
| C_15_H_24_O_2_-mono-TMS | 1815 | 308 | 0.08 | - | - | - | - | - | - | - | - | 0.40 | - |
| Caryophylla-3,8(13)-diene, 5,6-dihydroxy* | 1826 | 380 | 1.46 | - | - | - | - | - | - | 0.10 | 1.19 | 0.71 | - |
| NN (225,73,143) | 1836 | - | - | 0.21 | - | - | - | - | - | - | - | - | - |
| 5-Phenylpenta-2,4-dienoic acid | 1840 | 246 | - | - | 0.45 | - | - | - | - | - | - | - | 0.48 |
| NN (73, 143,210,147,275,290) | 1841 | - | 0.05 | - | - | - | - | - | - | - | - | - | - |
| Coniferyl aldehyde | 1844 | 250 | - | - | - | - | - | - | 0.07 | 0.09 | 0.42 | - | - |
| C_15_H_26_O_2_-mono-TMS (251,271,280) | 1849 | 310 | 0.13 | - | - | - | - | - | - | 0.06 | - | 0.07 | - |
| Tetradecanoic acid | 1851 | 300 | - | - | 0.05 | - | 0.04 | - | 0.03 | 0.14 | - | - | - |
| 2-Phenylethyl benzoate | 1856 | - | - | - | - | - | - | - | - | - | - | 0.27 | - |
| C_15_H_2_O_2_-mono-TMS | 1860 | 308 | 0.52 | - | - | - | - | - | - | - | 1.13 | 0.52 | - |
| NN (73>208,143,147,221) | 1870 | - | - | 0.05 | - | - | - | - | - | - | - | - | - |
| Caryophylla-4(12),8(13)-diene, 5,6-dihydroxy, isomer 1 | 1873 | 380 | 0.23 | - | - | - | - | - | - | - | 0.44 | 0.12 | - |
| Methyl *p*-coumarate | 1876 | - | - | - | - | - | - | - | - | 0.02 | - | - | - |
| C_15_H_24_O_2_-diTMS (73,169,147) | 1882 | 380 | 0.34 | - | - | - | - | - | - | - | 0.15 | 0.14 | - |
| C_15_H_26_O_2_-diTMS (73,193,295) | 1889 | 382 | - | 0.03 | - | - | - | - | - | - | - | - | - |
| C_15_H_24_O_2_-di-TMS(73,156,169) | 1895 | 380 | 0.12 | - | - | - | - | - | - | - | 0.15 | 0.07 | - |
| Sesquiterpenoid (73,208,147) | 1900 | - | 0.45 | - | - | - | - | - | - | 0.15 | - | 0.26 | - |
| Caryophylla-4(12),8(13)-diene, 5,6-dihydroxy, isomer 2* | 1902 | 380 | 2.70 | - | - | - | - | - | - | 0.57 | 2.13 | 0.94 | - |
| C_15_H_24_O_2_-diTMS (73,277,91) | 1921 | 380 | 0.73 | - | - | - | - | - | - | 0.02 | - | 0.08 | - |
| 14-Hydroxy-4,5-epoxycaryophyllene acetate | 1928 | 278 | 0.77 | - | - | - | - | - | - | - | 0.51 | 0.35 | - |
| (Z)-Ferulic acid | 1932 | 338 | - | - | - | - | - | - | 0.13 | 0.22 | 0.15 | - | - |
| *p*-Coumaric acid* | 1945 | 308 | 2.1 | trace | 1.5 | 3.0 | - | - | 5.90 | 6.73 | 5.50 | 2..62 | 9.77 |
| C15H26O-mono-TMS | 1951 | 294 | - | - | - | 2.50 | - |  |  |  | - |  | - |
| C_15_H_24_O_2_-mono-TMS | 1953 | 308 | 0.66 | - | - | - | - | - | - | - | - | 0.08 | - |
| C_15_H_24_O_2_-diTMS | 1983 | 380 | 0.39 | - | - | - | - | - | - | 0.35 | - | - | - |
| Gallic acid | 1984 | - | - | trace | - | - | 0.55 | - | - | - | - | - | - |
| Manool oxide | 1987 | 290 | - | - | - | - | - | 0.27 |  |  | - |  | - |
| 5,6,8-Trihydroxycaryolan* | 1989 | 470 | 2.85 | - | - | - | - | - | - | - | 2.38 | 1.46 | - |
| C_15_H_24_O_2_-di-TMS (73,147,157) | 1993 | 380 | - | - | - | - | - | - | - | - | - | - | - |
| (Z)-Caffeic acid | 2000 | 396 | - | - | - | - | - | - | 0.002 | 0.13 | - | - | - |
| Benzyl ester (91,73,285) | 2028 | 300 | - | - | 0.20 | - | - | - | 0.35 | 0.23 | - | 0.22 | - |
| 3,4-Dimethoxycinnamic acid* | 2031 | 280 | - | - | - | 5.26 | - | - | - |  | - | - | 0.62 |
| Isobutyl (E)-*p*-coumarate | 2037 | 292 | - | - | - | - | - | - | - | - | - | 0.13 | - |
| Palmitelaidic acid | 2040 | 326 | - | - | - | - | 0.04 | - | - | - | - | - | - |
| 3-Hydroxymyristic acid | 2041 | - | - | - | 0.02 | - | trace | - | 0.08 | 0.04 | trace | 0.03 | - |
| Hexadecanoic acid | 2048 | 328 | 0.85 | 0.20 | 0.05 |  | 0.11 | 0.63 | 0.22 | 0.17 | 0.76 | 0.29 | 0.17 |
| C_15_H_24_O_2_-diTMS | 2059 | 380 | 0.28 | - | - | - | - | - | - | - | - | 0.06 | - |
| Benzyl cinnamate * | 2085 | 238 | - | - | 2.02 | - | - | - | 0.45 | 0.66 | 0.75 | 0.12 | - |
| Isoferulic (hesperetic) acid* | 2089 | 338 | - | - | - | 4.25 | - | - | - | - | 0.8 | - | 0.54 |
| *n-*Heneicosane | 2100 | - | 0.13 |  | - | - | - | 0.02 | - | - | - | - | - |
| (E)-Ferulic acid* | 2103 | 338 | trace | - | 2.19 | 1.10 | - | - | 4.29 | 5.81 | 4.3 | 0.40 | 0.53 |
| 3,4-Dihydroxycimmamyl alcohol? | 2124 | - | - | - | - | - | - | - | - | 0.03 | - | - | - |
| NN (73,191,147) | 2129 | - | 0.22 | - | - | - | - | - | - | - | - | 0.04 | - |
| NN (73,156,157) | 2140 | - | - | - | - | - | - | - | - | - | - | 0.11 | - |
| 3-Methylbutanyl (E)-*p*-coumarate | 2151 | 306 | - | - | - | 0.81 | - | - | - | - | - | 0.55 | - |
| Caffeic acid* | 2155 | 396 | trace | - | 1.79 | 3.30 | - | - | 0.58 | 0.73 | 0.03 | 0.10 | 1.67 |
| 3-Methyl-3-butenyl (E)-*p*-coumarate | 2160 | 304 | - | - | - | 0.05 | - | - | - | - | - | 0.05 | 0.14 |
| Phytol | 2183 | - | - | - | - | - | - | 0.12 | - | - | - | - | - |
| 3-Oxomanoyl oxide? | 2192 | - | - | - | - | - | - | 0.48 | - | - | - | - | - |
| 2-Methyl 2-butenyl (E)-*p*-coumarate | 2205 | 304 | - | - | - | 0.25 | - | - | - | - | - | 0.60 | 0.03 |
| 3-Methyl 2-butenyl (E)-*p*-coumarate* | 2211 | 304 | - | - | - | 0.88 | - | - | - | - | trace | 0.05 | 0.14 |
| Linoleic acid | 2215 | 352 | 0.02 | 1.03 | 0.03 | trace | - | 0.15 | - | - | - | - | - |
| α-Linolenic acid | 2219 | 350 | - | 1.48 | - | - | - | - | - | - | - | - | - |
| Oleic acid | 2219 | 354 | trace | 0.11 | 0.04 | trace | 0.04 | 0.33 | 0.13 | 0.14 | 0.71 | 0.10 | 0.05 |
| 3-Hydroxyhexadecanoic acid | 2234 | - | - | - | trace | - | - | - | - | 0.02 | - | - | - |
| Octadecanoic acid | 2246 | 356 | 0.12 | 0.05 | trace | 0.05 | 0.11 | 0.11 | 0.06 | 0.06 | trace | 0.03 | trace |
| Diterpenoid? (73,363,81) | 2259 | - | - | - | - | - | - | 0.37 | - | - | - | - | - |
| Diterpenoid? (298,283,165) | 2261 | - | - | - | - | - | - | 0.66 | - | - | - | - | - |
| *n-*Hexyl *p*-coumarate | 2292 | 320 | 0.06 | - | - | - | - | - | - | - | - | trace | - |
| 3-Methyl-3-butenyl-4-acetyloxy (E)-caffeate | 2294 | - | - | - | - | - | - | - | - | - | - | 0.03 | - |
| *n*-Tricosane | 2300 | - | 0.47 | 0.09 | 0.02 | trace | 0.18 | - | 0.14 | 0.15 | 0.67 | 0.30 | 0.07 |
| 3-Methyl-2-butenyl (E)-ferulate | 2303 | 334 | - | - | - | 0.02 | - | - | - | - | - | - | - |
| Sandaracopimaric acid | 2316 | 374 | - | - | - | - | - | 0.37 | - | - | - | - | - |
| 3-Methyl-3-butenyl (E)-ferulate | 2317 | 334 | - | - | - | 0.10 | - | - | - | - | - | - | trace |
| Benzyl (Z)-*p*-coumarate | 2322 | 326 | - | - | - | - | - | - | 0.31 | 0.32 |  | 0.02 | - |
| *n*-Butanyl (E)-caffeate | 2325 | - | - | - | - | trace | - | - | - | - |  | - | - |
| Isopimaric acid* | 2336 | 374 | - | - | - | - | - | 2.62 | - | - | - | - | - |
| Palustric acid* | 2356 | 374 | - | - | - | - | - | 0.93 | - | - | - | - | - |
| 3-Methylbutanyl (E)-caffeate | 2357 | - | - | - | - | trace | - | - | - | - | - | 0.01 | - |
| 1-Eicosanol | 2360 | - | - | 0.11 | trace | - | 0.04 | - | - | - | - | 0.07 | - |
| NN (350,235,73) | 2365 | - | - | - | - | - | - | - | 0.07 | 0.05 | - | - | - |
| 3-Methyl-3-butenyl caffeate* | 2365 | 392 | - | - | - | 1.11 | - | - | - | - | 0.02 | - | 2.20 |
| Pimaric acid* | 2378 | 374 | - | - | - | - | - | 3.63 | - | - | - | - | - |
| 2-Myristate | 2385 | - | - | - | - | - | 0.03 | - | - | - | - | - | - |
| Dehydroabietic acid* | 2389 | 372 | - | - | - | - | - | 3.36 | - | - | - | 0.03 | - |
| *n-*Tetracosane | 2400 | - | - | - | - | trace | 0.01 | - | - | - | - | 0.06 | - |
| 2-Methyl-2-butenyl (E)-caffeate | 2413 | 392 | - | - | - | 0.27 | - | - | - | - | - | 0.04 | 0.41 |
| Abietic acid* | 2414 | 374 | - | - | - | - | - | 1.52 | - | - | - | - | - |
| 2’,6’-Dihydroxy-4’-mathoxydihydrochalcone | 2418 | 416 | - | - | - | 0.18 |  | - | - | - | - | 0.36 | 0.09 |
| 1-Myristate | 2418 | - | - | - | - | - | 0.07 | - | - | - | - | - | - |
| 3-Methyl-2-butenyl (E)-caffeate* | 2422 | 392 | - | - | - | 1.33 | - | - | - | - | 0.03 | - | 3.33 |
| 3-Hydroxyoctadecanoic acid | 2428 | - | - | - | - | - | 0.06 | - | - | 0.03 | - | - | - |
| Eicosanoic acid | 2445 | 384 | 0.17 | 0.03 | 0.01 | - | 0.28 | 0.16 | 0.04 | 0.06 | 0.21 | 0.05 | - |
| 2’,4’,6’-Trihydroxydihydrochalcone | 2455 | 474 | - | - | - | 0.45 | - | - | - | - | - | - | 0.18 |
| Benzyl (Z)-ferulate | 2457 | 356 | - | - | 0.23 | - | - | - | 0.16 | 0.19 | trace | 0.10 | - |
| 1-Heneicosanol | 2458 | - | - | - | - | - | 0.02 | - | - | - | - | - | - |
| Pinocembrin, mono-OTMS | 2458 | 328 | - | - | - | 0.70 | - | - | - | - | - | - | - |
| Diterpene acid (73,121,256) | 2461 | 374 | - | - | - | - | - | 5.38 | - | - | - | - | - |
| 4-Hydroxycinnamyl benzoate | 2476 | 326 | - | - | - | - | - | - | 1.70 | 0.96 | - | 0.08 | - |
| 2’,6’,a-Trihydroxy-4’-methoxychalcone | 2492 | 502 | - | - | - | 0.35 | - | - | - | - | - | - | 0.19 |
| *n*-Pentacosane | 2500 | - | 1.25 | 1.50 | - | 0.25 | 0.25 | - | 0.52 | 0.55 | 1.49 | 0.57 | 0.31 |
| Pinostrobin chalcone* | 2507 | 414 | - | - | - | 4.25 | - | - | - | - | - | 0.49 | 0.09 |
| Neoabietic acid* | 2508 | 374 | - | - | - | - | - | 11.9 | - | - | - | - | - |
| Pinostrobin | 2512 | - | - | - | - | 0.68 | - | - | - |  |  | 020 | 0.28 |
| Benzyl (E)-*p*-coumarate* | 2516 | 326 | - | - | - | 0.66 | - | - | 6.93 | 6.55 | 3.46 | 0.91 | 3.24 |
| 1-*p*-Coumaroylglycerol | 2525 | 454 | - | - | 0.15 | - | - | - | 0.08 | 0.10 | - | 0.01 | - |
| NN (105,77,131) | 2530 | - | - | - | - | - | - | - | 0.07 | 0.07 | - | - | - |
| Pinocembrin chalcone* | 2542 | 472 | - | - | - | 4.93 | - | - | - | - | - | - | 0.18 |
| Phthalate | 2546 | - | - | - | - | - | - | - | 0.23 | - | 0.63 | - | - |
| Pinocembrin* | 2549 | 400 | - | - | - | 5.20 | - | - | - | - | - | 1.24 | 10.96 |
| 1-Docosanol | 2556 | - | 0.33 | 1.20 | 0.28 | - | 1.83 | 0.27 |  |  | - | 0.09 | - |
| 1-Acetyl-2-*p*-coumaroylglycerol | 2557 | 424 | - | - | 0.17 | - | - | - | 0.04 | 0.06 | trace | - | - |
| Diterpenoid C_26_H_44_O_3_Si_2_ | 2561 | 460 | - | - | - | - | - | 1.88 | - | - | - | - | - |
| 2-Acetyl-1-*p*-coumaroylglycerol | 2577 | 424 | - | - | 0.17 |  |  | - | 0.19 | 0.23 |  | - | - |
| α-Glyceryl palmitelaidate | 2592 | - | - | - | - | - | 0.03 | - | - | - | - | - | - |
| Diterpenoid (121,73,309) | 2599 | 422 | - | - | - | - | - | 27.6 | - | - | - | - | - |
| 2-Phenylethyl (E)-*p*-coumarate | 2600 | 340 | 0.05 | - | - | 0.73 | - | - | 0.08 | 0.05 | - | 0.46 | 0.21 |
| NN (105,189,73,143) | 2606 | - | - | - | 0.13 | - | - | - | 0.12 | 0.06 | - | - | 0.57 |
| Pinobanksin* | 2609 | 488 | - | - | - | 1.62 | - | - | - | - | - | 0.22 | 2.85 |
| 1-Monopalmitin | 2610 | - | - | - | - | - | 0.05 | - | - | - | - | - | - |
| Diterpenoid (75,239,238) | 2613 | 462 | - | - | - | - | - | 2.38 | - | - | - | - | - |
| 1,2-Diacetyl-3-*p*-coumaroyl glycerol | 2618 | 394 | - | - | 0.10 | - | - | - | 0.09 | 0.10 | - | - | - |
| Coniferyl benzoate* | 2639 | 356 | - | - | 0.57 | 1.04 | - | - | 11.96 | 14.76 | 0.36 | 0.34 | - |
| Docosanoic acid | 2642 | 412 | 0.40 | 0.08 | 0.08 | - | 0.16 | - | 0.17 | 0.22 | 0.82 | 0.35 | 0.12 |
| Pinifolic acid* | 2646 | 480 | - | - | - | - | - | 11.5 | - | - | - | - | - |
| 1-Tricosanol | 2655 | - | - | - | - | - | 0.04 | - | - | - | - | - | - |
| Benzyl (E)-isoferulate | 2656 | 356 | - | - | - | 0.66 | - | - | - | - | - | - | - |
| 1-Feruloyl glycerol | 2670 | 484 | - | - | 0.07 | - | - | - | - | - | - | - | - |
| Chalcone (192,311,73) | 2673 | 430 | - | - | - | 1.54 | - |  |  |  | - |  | 0.54 |
| Benzyl (E)-ferulate* | 2680 | 356 | - | - | 0.73 | 0.76 | - | - | 3.04 | 3.80 | trace | 0.37 | 0.29 |
| Pinobanksin 3-acetate* | 2690 | 430 | - | - | - | 8.21 | - | - | - | - | - | 0.54 | 8.84 |
| *n*-Heptacosane | 2695 | - | 0.49 | 1.92 | 0.51 | 0.47 | 0.98 | - | 1.71 | 1.75 | 4.61 | 1.93 | 1.83 |
| 1-Caffeoyl glycerol | 2708 | 542 | - | - | 0.27 | - | - | - | - | - |  | - | - |
| Benzyl (E)-caffeate* | 2723 | 414 | - | - | 1.13 | 6.33 | - | - | 0.73 | 0.65 | 0.32 | 0.07 | 2.60 |
| 7,15-Dihydroxydehydroabietic acid* | 2728 | 548 | - | - | - | - | - | 3.37 | - | - | - | - | - |
| 2-Acetyl-1-caffeoylglycerol | 2732 | 512 | - | - | 0.13 | - |  | - | 0.03 | 0.04 | trace | - | - |
| 1-Monolinoleoyl glycerol | 2739 | 498 | - | - | - | - | 0.61 | - | - | - | -- | - | -- |
| Isoalpinin | 2740 | 428 | - | - | 1.17 | - | - | - | 0.24 | 0.25 | - | 0.49 | - |
| 2-Monooleoyl glycerol | 2744 | - | - | - | - | - | 0.23 | - | - | - | - | - | - |
| Chrysin* | 2748 | 398 | - | - |  | 4.72 | - |  |  |  | trace |  | 5.62 |
| 1-Tetracosanol | 2750 | - | 0.20 | 0.37 | 0.31 | 0.13 | trace | 0.23 | - | 0.03 | trace | 0.08 |  |
| 1-Acetyl-3-caffeoylglycerol | 2760 | 512 | - | - | 0.49 | - | - | - | 0.07 | 0.09 |  | - |  |
| Diterpenoid (75,156,302) | 2760 | 420 | - | - | - | - | - | 2.29 | - | - | - | - | - |
| Galangin* | 2766 | 486 | - | - | - | 5.18 | - | - | - | - | - | 0.49 | 6.82 |
| NN (179,192,358) | 2773 | - | 0.23 | - | - | - | - | - | 0.06 | 0.27 | 1.12 | 0.47 | - |
| 1-Monolinonoleoyl glycerol* | 2775 | 498 | - | - | - | - | 1.27 | - | - | - | - | - | - |
| 1-Monooleoyl glycerol* | 2780 | 500 | - | - | - | - | 2.07 | - | - | - | - | - | - |
| Benzyl stearate | 2782 | 374 | - | - | - | - | - | - | - | 0.02 | - | - | - |
| NN (357,283,73) | 2788 | - | - | - | - | - | - | - | - | 0.16 | - | - | - |
| Pinobanksin 3-isobutanoate | 2790 | 486 | - | - | - | 0.25 | - | - | - | - | - | - | 0.13 |
| 5-Hydroxy-4’,7-dimethoxyflavanone* | 2793 | 372 | 2.12 | - | - | - | - | - | - | - | 1.18 | 0.49 | - |
| *n*-Octacosane | 2800 | - | - | 0.01 | - | - | - | - | - | 0.03 | - | - | - |
| 1,2-Diacetyl-3-caffeoyl glycerol | 2801 | 482 | - | - | 0.37 | - | - | - | - | - | - | - | - |
| 2-Phenylethyl-(E)-caffeate * | 2805 | 428 | - | - |  | 3.67 | - | - | - | - | - | 0.08 | 1.59 |
| NN (201,55,83) | 2808 | - | - | - | 0.16 |  | - | - | - | - | - | - | - |
| 1-Monostearin* | 2808 | - | - | - | - | - | 3.30 | - | - | - | -- | - | - |
| Chalcone? (201,55,83) | 2822 | 444 | - | - | - | 0.30 | - | - | - | 0.09 | - | - | - |
| Isosakuranetin | 2817 | 430 | 0.92 | - | 0.47 | - | - | - | 0.06 | 0.30 | 0.45 | 1.81 | 0.91 |
| NN (487,474,73) | 2825 | 502 | - | - | - | - | - | - | - | - | - | - | - |
| Chalcone (222,73,311) | 2830 | 460 | 0.16 | - | - | - | - | - | - | - | - | - | - |
| Caffeate (73,396,307) | 2830 | - | - | - | - | - | - | - |  | 0.18 | - | - | - |
| Cinnamyl (E)-*p*-coumarate* | 2837 | 352 | - | - | - | 1.02 | - | - | - | - | - | 0.91 | 3.31 |
| Hexacosanal | 2837 | - | trace | 0.02 | - | - | - | - | - | - | - | - | - |
| Tetracosanoic acid | 2842 | 440 | 0.38 | 0.19 | 0.03 | - | - | 0.19 | 0.52 | 1.70 | 1.75 | 2.21 | 5.44 |
| 2-Monononadecanoyl glycerol* | 2867 | - | - | - | - | - | 1.46 | - | - | - | - | - | - |
| Sakuranetin chalcone | 2868 | 502 | trace | - | - | trace | - |  | - | 0.05- |  | 0.43 | - |
| Sakuranetin * | 2879 | 440 | 18.6 | 0.03 | - | trace | - | 0.14 | 2.07 | 3.45 | 9.68 | 10.29 | 0.16 |
| Diterpenoid (73,121,143) | 2884 | - | - | - | - | - | -- | 3.57 | - | - | - | - | - |
| Pinobanksin 3-pentanoate | 2882 | - | - | - | - | 0.17 | - | - | - | - | - | - | 0.31 |
| NN (193,73,205) | 2886 | - | - | - | - | - | - | - | 0.04 | 0.34 | - | - | - |
| Naringenin* | 2891 | 488 | - | - | 0.32 | 0.02 | - | - | 0.06 | 0.21 | 1.29 | 0.76 | 0.15 |
| *n*-Nanocosane | 2900 |  | 0.15 | 0.12 | - | 0.14 | 2.42 | - | 0.80 | 0.74 | 0.56 | 0.97 | 0.90 |
| 1-Monononadecanoyl glycerol* | 2904 | - | - | - | - | - | 2.04 | - | - | - | - | - | - |
| Chalcone (280,73,311) | 2915 | 518 | 1.14 | - | - | - | - | - | 0.15 | 0.09 | 1.03 | 0.78 | - |
| Triterpenoid (189,73,129) | 2927 | 512 | - | - | 0.39 | - | - | - | - | 0.21 | - | - | - |
| Hydrocinnamyl caffeate | 2931 | 442 | - | - | - | 0.32 | - | - | - | - | - | - | - |
| Triterpenoid (189,73,129) | 2934 | - | - | - | 0.15 | - | - | - | 0.11 | 0.21 | - | - | - |
| Catechine* | 2936 | 650 | trace | 0.24 | - | - | 1.03 | 1.90 | - | - | - | - | - |
| NN (73,69,81,517) | 2943 | - | - | - | - | - | - | - | - | - | - | - | - |
| 1-Hexacosanol | 2948 | - | - | 0.11 | 0.16 | trace | 0.89 | - | - | - | - | - | - |
| NN (371,73,328,386) | 2960 | - | - | - | - | - | - | - | - | 0.04 | 0.34 | - | - |
| 2-Monoarachdate* | 2964 | 530 | - | - | - | - | 15.7 | - | - | - | - | - | - |
| (E)-Cinnamyl-(E)-isoferulate * | 2973 | 382 | - | - | - | 2.61 | - | - | 0.34 | 0.26 | - | - | 0.09 |
| Hesperetin | 2978 | 518 | - | - | 0.03 | - | - | - | - | - | - | - | - |
| Triterpenoid? (73,247,426) | 2985 | - |  | - | - | - | - | - | 0.26 | 0.35 | - | - | - |
| Acacetin, mono-TMS | 2988 | 356 | - | - | 0.53 | - | - | - | 0.24 | 0.39 | 0.94 | 0.71 | - |
| Glyceryl eicosenoate | 2985 | 528 | - | - | - | - | 0.10 | - | - | - | - | - | - |
| 1-Monoarachidate* | 3000 | 530 | - | - | - | - | 2.92 | - | - | - | - | - | - |
| γ-Tocopherol | 3010 | 488 | - | - | - | - | 0.41 | - | - | - | - | - | - |
| 3,5,7-Trihydroxy-4'-methoxyflavone, di-TMS* | 3015 | 444 | 1.27 | trace | 0.31 | - | - | - | 0.34 | 0.35 | 1.91 | 0.82 | - |
| NN (356,341,310) | 3023 | - |  | - | 0.07 | - | - | - | - | - | - | - | - |
| Flavonoid (443,444,73) | 3031 | 458 | 0.15 | - | - | - | - | - | 0.74 | 0.09 | 2.12 | 3..51 | - |
| Unidentified glyceride 1 | 3031 | - | - | - | - | - | 3.54 | - | - | - | - | - | - |
| Piloin* | 3035 | 458 | 3.25 | - | - | - | - | - | - | 1.26 | 2.12 | - | - |
| Cinnamyl (E)-caffeate | 3040 | 440 | - | - | - | 4.66 | - |  | - | - | - | - | 2.07 |
| Hexacosanoic acid | 3040 | 468 | 0.58 | 1.22 | 0.08 | - | - | - | - | - | 1.01 | 1.00 | - |
| Homoeriodictyol * | 3048 | 516 | 4.92 | - | - | - | - | - | 0.15 | 0.30 | 2.74 | 3.38 | - |
| Unidenifoed glycerides 2 | 3053 | - | - | - | - | - | 0.87 | - | - | - | - | - | - |
| Pectolinaringenin* | 3060 | 45 | 7.49 | - | - | - | - | -- | - | 0.61 | 2.73 | 5.07 | - |
| Acacetin* | 3065 | 428 | 1.59 | 0.02 | 0.06 | - | - | - | - | 0.36 | 1.80 | 1.85 | - |
| 9-Hentriacontene | 3071 | - | - | - | - | - | - | - | 0.30 | 0.28 | 1.33 | - | 0.08 |
| 7-Hentriacontene | 3078 | - | - | - | - | - | - | - | 0.37 | 0.44 | 1.16 | - | 0.12 |
| NN (414,73,399,192) | 3079 | - | - | 0.06 | 0.23 | - | - | - | - | trace | - | - | - |
| 1,3-Diacetyl-2-arachidate* | 3081 | 470 | - | - | - | - | 1.59 | - | - | - | - | - | - |
| NN (443,403,73) | 3091 | - | - | 0.04 | - | - | - | - | - | 0.37- | 0.37 | - | - |
| Kaempheride* | 3099 | 516 | 4.78 | - | - | - |  | - | - | 2.94 | 2.94 | 3.40 | 0.67 |
| *n*-Hentriacontane | 3100 | - | - | - | - | - | trace | - | 0.38 | trace | - | - | trace |
| Kaempherol * | 3114 | 574 | 2.68 | 0.04 | trace | - | - | - | 0.07 | 0.87 | 0.87 | 1.59 | 0.52 |
| Dimethylquercetin | 3120 | 546 | 0.62 | 1.12 | - | - | 6.15 | - | 0.18 | 0.19 | 0.17 | 0.51 | - |
| NN (189,73,105) | 3121 | - |  | - | 0.56 | - | - | - | - | - | - | - | - |
| Trimethylquercetin* | 3131 | 488 | 1.75 | 0.65 | - | - | 2.08 | - | - | 0.51 | 0.51 | 1.18 | - |
| 6-Hydroxycaryophyllene coumarate * | 3136 | 438 | 4.78 | - | - | - | - | - | 0.20 | 0.38 | 3.21 | 1.35 | - |
| NN (73,247,456) | 3139 | - | - | - | - | - | - | - | 0.35 | 0.63 | - | - | - |
| 5,7,4'-Trihydroxy-3-methoxyflavone | 3140 | 516 | - | 0.36 | - | 0.13 | - | - | - | - | - | 0.67 | 0.27 |
| 1-Octacosanol | 3146 | - | - | - | - | - | - | - | - | - | trace | - | - |
| Apigenin | 3157 | 486 | 0.08 | - | 0.11 | 0.23 | - | - | 0.23 | 0.31 | 0.35 | 0.58 | 0.23 |
| 2-Monobechenin* | 3159 | 558 | - | - | - | - | 1.09 | - | - | - | - | - | - |
| Triterpenoid (107,175,147) | 3162 | 512 | - | - | - | - | - | - | - | - | - | - | 0.04 |
| 14-Hydroxyβ-caryophyllene *p*-coumarate* | 3165 | 438 | 3.72 | - | - | - | - | - | 0.33 | 0.43 | 1.13 | 0.73 | -- |
| Triterpenoid (189,73,501) | 3167 | - | - |  |  | 0.47 | - |  |  |  | - |  | -- |
| NN (267,73,425) | 3169 | - | - | - | - | - | - | - | 0.11 | 0.23 | - | - | -- |
| NN (459,460,429) | 3173 | - | - | - | - | - | - | - | - | - | - | - | - |
| Triterpenoid (189,73,572) | 3179 | - | - | - | - | 0.60 | - | - | - | - | - | - | - |
| 14-Hydroxy-β-isocaryophyllene *p*-coumarate * | 3180 | 438 | 0.39 | - | - | - | - | - | 0.16 | 0.15 | 0.45 | 0.81 | - |
| Triterpenoid (73,147,175) | 3183 | 512 |  | 0.38 | - | - | - | - | - | - | - | - | - |
| C_15_H_24_O *p*-coumarate (219,236) | 3186 | 438 | 0.88 | - | - | - | - | - | - | - | 0.27 | 0.68 | - |
| 1-Monobechenin* | 3195 | - | - | - | - | - | 1.74 | - | - | - | - | - | -- |
| Quercetin, dimethyl ether, isomer 2 | 3206 | 546 | 0.54 | - | - | - | 0.61 | - | - | - | 0.45 | 0.56 | - |
| NN (297,73,105) | 3212 | - | - | - | - | - | - | - | 0.21 | 0.12 | - | - | - |
| Triterpenoid (189) | 3226 | - | 1.43 | - | - | - | - | - | - | - | - | 0.72 | - |
| Caffeate (267,73,396) | 3229 | - | - | - | - | - | - | - | 0.29 | 0.39 | 0.28 | - | - |
| Isorhamnetin* | 3241 | 614 | 4.59 | - | - | - | - | - | 0.15 | 0.15 | 1.94 | 1.86 | - |
| Octacosanoic acid | 3243 | 496 | - | 0.71 | - | - | - | - | 0.05 | 0.15 | trace | - | 1.77 |
| C_15_H_26_O *p*-coumarate (219,205,73) * | 3244 | 440 | 0.16 | - | - | - | - | - | 2.26 | 3.95 | trace | 1.86 | - |
| Triterpenoid (131,74,144) | 3252 | 502 | - | - | 0.25 | - | - | - | - | 0.18 | 0.36 | - | - |
| Triterpenoid (367,69,73) | 3255 | 512 | - | 0.43 | - | - | - | - | - | - | - | - | - |
| Triterpenoid (170,73,397) | 3264 | 523 | - | - | - | 0.26 | - | - | - | - | - | - | - |
| Quercetin dimethyl ether, isomer 3 | 3269 | 546 | 0.68 | - | - | - | - | - | - | - | - | 0.57 | - |
| 9-Tritriacontene | 3273 | - | - | - | - | - | - | - | 1.27 | 1.87 | 0.15 | 1.37 | - |
| Triterpenoid (109,73,69) * | 3278 | 512 | - | 14.27 | 0.10 | - | - | - | - | - | - | - | - |
| 6-Hydroxy-β-caryophyllene ferulate | 3279 | 468 | 0.14 | - | - | - | - | - | - | trace | trace | - | - |
| Diacetylglyceride | 3281 | - | - | - | - | - | 1.06 | - | - | - | - | - | - |
| Benzyl ester? (105,237,73) | 3295 | 566 | 0.16 | - | - | - | - | - | 0.91 | 0.69 | 1.11 | - | - |
| *n*-Tritriacontane | 3300 | - | trace | trace | - | - | - | - | - | - | - | - | - |
| Quercetin methyl ether + *p*-coumarate? | 3301 | - | 0.18 | - | - | - | - | - | - | - | - | 0.33 | - |
| Triterpenoid (189,69,175) | 3304 | 512 |  | 0.83 | - | 0.20 | - | - | - | - | - | - | - |
| 14-Hydroxy-β-caryophyllene ferulate* | 3305 | 468 | 0.88 | - | - | - | - | - | 0.06 | 0.002 | 0.43 | 0.13 | - |
| Dammaradien-3-one* | 3310 | 424 | - | 4.71 | - | 0.48 | 1.35 | - | - | - | - | - | - |
| 14-Hydroxyisocaryophyllene ferulate | 3312 | 468 | 0.10 | - | - | - | - | - | 0.08 | 0.02 | trace | - | - |
| Triterpenoid (109,73,512) * | 3323 | 512 | - | 1.50 | - | - | - | - | - | - | - | - | - |
| C_15_H_24_O ferulate (219,236,249) | 3330 | 468 | 0.12 | - | - | - | - | - | - | - | - | 0.13 | - |
| Triterpenoid (69,109,73) | 3333 | - | - | - | - | 0.02 | - | - | - | - | - | - | - |
| 1-Triacontanol | 3344 | - | - | - | - | - | 0.50 | - | 0.22 | 0.15 | - | 0.24 | 0.08 |
| β-Sitosterol | 3347 | 486 | 0.21 | - | - | - | - | 2.52 | - | - | 0.05 | - | - |
| NN (223…502) | 3353 | - |  | - | 0.16 | - | - | - | 0.15 | - | - | - | - |
| 14-Hydroxy-β-caryophyllene caffeate* | 3360 | 438 | 0.22 | - | - | - | - | - | 0.48 | - | - | 0.33 | - |
| Triterpenoid | 3368 | - | - | - | - | 0.52 | - |  | - |  | - |  | - |
| Triterpenoid* | 3382 | - | - | 1.41 | - | - | - | - | - | - | trace | - | - |
| Coniferyl *p*-coumarate * | 3403 | 470 | 0.35 | - | - | - | - | - | 1.41 | 2.48 | 0.12 | 0.30 | - |
| Ferulate (249,205,470,219) | 3407 | - | 0.23 | - | - | - | - | - | 0.88 | 0.75 | - | - | - |
| Triterpenoid (199,73,117) * | 3419 | - | - | 2.49 | - | - | - | - | - | - | 0.18 | - | - |
| Triterpenoid (189,73,69) | 3424 | - | 0.23 | - | - | - | - | - | - | - | - | 0.12 | - |
| Triterpenoid (199,73,117,145) | 3438 | - | - | - | 0.61 | - | 0.50 | - | 0.51 | 0.39 | - | - | - |
| Triacontanoic acid | 3441 | 524 | - | - | 0.25 | - | - | - | - | - | 0.12 | 0.48 | 1.13 |
| Triterpenoid (109,121,123) * | 3442 | 512 | - | 2.64 | - | - | - | - | - | - | - | - | 0.06 |
| Triterpenoid (199,69,519) * | 3448 | - | - | 5.91 | - | - | -- | - | - | - | - | - | - |
| Triterpenoid (109,69,79) * | 3468 | - | - | 1.62 | - | 0.17 | - | - | - | - | - | - | - |
| Triterpenoid (109,73,69) * | 3490 | 512 | - | 3.98 | - | - | - | - | - | - | - | - | - |
| Triterpenoid | 3495 | - | - | - | - | 0.15 | 1.50 |  | - | - | - | - | - |
| Triterpenoid (199,69,73) | 3503 | 512 | - | 2.09 | 0.49 | - | - | - | 0.95 | 0.66 | 0.12 | - | - |
| Dipterocarpol* | 3510 | 514 | trace | 33.0 | - | - | 1.17 | - | - | - | - | - | - |
| Triterpenoid (108,69,257) | 3519 | - | - | - | - | - | 0.89 | - | 0.53 | 0.10 | - | - | - |
| Triterpenoid (108,357,422) * | 3529 | - | - | 2.59 | - | - | - | - | - | - | - | - | - |
| Triterpenoid (109,69,73) | 3532 | - | 0.08 | - | - | - | 4.05 | - | - | - | - | - | - |
| 1-Dotriacontanol | 3538 | - | - | - | - | - | - | - | - | - | - | 0.40 | 0.09 |
| Triterpenoid (203,189,73) | 3543 | - | - | - | 0.39 | - | - | - | 0.73 | 0.67 | - | - | 0.35 |
| Triterpenoid (199,189,73) * | 3544 | - | - | 4.98 | - | - | - | - | - | - | - | - | - |
| Octadecyl hexacosanoate | 3550 | 508 |  | - | - | - | - | - | - | - | - | - | 0.22 |
| Triterpenoid (199,69,422) | 3554 | 528 | - | 1.98 | - | - | - | - | - | - | - | - | - |
| Coniferyl ferulate | 3560 | 500 | 0.09 | - | - | - | - | - | 1.29 | 2.88 | 0.21 | 0.35 | - |
| NN (393,73,139) | 3561 | - | - | - | - | 0.28 | - | - | - | - | - | - | - |
| Triterpenoid (108,199,69) * | 3570 | - | - | 2.16 | - | - | - | - | - | - | - | - | - |
| Oleanoic acid (203,73,320) | 3574 | 600 | - | - | - | - | - | - | 0.26 | - | - | - | - |
| Coniferyl caffeate | 3583 | 558 | - | - | - | - | - | - | 0.38 | 0.28 | - | - | - |
| Triterpenoid (73,109,203) * | 3585 | - | - | 2.12 | - | - | - | - | - | - | - | - | - |
| Triterpenoid (189,73,117) | 3588 | - | - | - | - | - | - | - | - | 0.19 | - | - | - |
| NN (197,73) | 3610 | - | - | - | - | - |  | - | - | - | - | 0.41 | - |
| Triterpenoid (131,215,73) * | 3613 | 512 | - | 1.41 | - | - | - | - | - | - | - | - | - |
| Triterpenoid (131,215,73) * | 3620 | 512 | - | 1.69 | - | - | - | - | - | - | - | - | - |
| Triterpenoid (197,143,107) * | 3628 | - | 0.12 | 1.07 | - | - | - | - | - | - | - | - | - |
| Dotriacontanoic acid | 3635 | 552 | - | - | - | - | - | - | 0.26 | 0.19 | - | 0.30 | 0.99 |
| Triterpenoid (199,69,73) * | 3664 | - | - | 2.29 | - | - | - | - | - | - | - | - | - |
| Triterpenoid (431,143,117) | 3683 | - | - | 1.55 | - | - | - | - | - | - | - | - | - |
| Triterpenol acetate? (108,109,43) | 3702 | - | - | - | - | - | - | - | 0.58 | 0.22 | - | - | - |
| Triterpenoid (108,109,135)* | 3710 | - | - | 2.52 | - | - | - | - | - | - | - | - | - |
| NN (297,73,104,105) | 3714 | - | - |  | - | - | - | - | 0.61 | 0.41 | - | - | - |
| 2-Phenylethyl hexacosanoate | 3722 | - | - | 0.50 | - | - | - | - | - | - | - | - | - |
| Triterpenoid (131,215,189) | 3725 | - | - | 1.07 | - | - | 2.95 | - | - | - | - | - | - |
| 1-Acetyl-2,3-di-*p*-coumarate | 3726 | - | - | - | 0.75 | - | - | - | - | 0.36 | - | - | - |
| Triterpenoid (199,69,489)* | 3736 | - | - | 1.92 | - | - | 1.14 | - | - | - | - | - | - |
| Triterpenoid (131,215,73) | 3748 | - | - | 0.85 | - | - | - | - | - | - | - | - | - |
| Triterpenoid (199,69,73)* | 3763 | - | - | 1.67 | - | - | 3.02 | - | - | - | - | - | - |
| Tetratriacontanoic acid | 3839 | - | - | - | - | - | - | - | - | - | - | 0.54 | 1.76 |
| NN (297,73,203) | 3842 | - | - | - | - | - | - | - | 0.94 | 1.12 | - | - | - |
| Triterpenoid (131,215,73,389) | 3850 | - | - | 0.87 | - | - | - | - | - | - | - | - | - |
| Triterpenoid (131,215,73,389) | 3859 | - | - | 0.93 | - | - | - | - | 0.36 | - | - | - | - |
| 1,3-di-*p*-Coumaroyl glycerol* | 3870 | 600 | - | - | 2.83 | - | - | - | 0.69 | 0.13 | 0.49 | 0.86 | 0.20 |
| Triterpenoid (131,215,357) | 3876 | - | - | 0.46 | - | - | - | - | 0.28 | - | - | - | - |
| Triterpenoid (131,215,73) | 3898 | - | - | 0.45 | - | - | - | - | - | - | - | - | - |
| Triterpenoid (189,129,73) * | 3902 | - | - | - | 1.25 | - | - | - | - |  | - | - | - |
| Triterpenoid (189,129,73) * | 3915 | - | - | - | 1.22 | - | - | - | - |  | - | - | - |
| Docosyl *p*-coumarate (219,236) | 3933 | 438 | 0.47 | - | - | - | - | - | - | - | - | 0.15 | - |
| Triterpenoid (189,73,129) | 3934 | - | - | - | - | - | - | - | 0.47 | 0.71 | - | - | - |
| 2-Acetyl-1,3-di-*p*-comaroyl glycerol* | 3960 | 570 | - | - | 10.5 | - | - | - | 3.83 | 3.39 | 2.85 | 1.66 | 1.22 |
| Docosyl hexadecanoate | 3965 | 564 | - | 1.05 | - | - | - | - |  | - | - | - | - |
| 2-*p*-Coumaroyl-1-feruloyl glycerol | 3980 | 630 | - | - | 0.42 | - | - | - |  | 0.13 | - | - | - |
| 1-*p*-Coumaroyl-3-feruloyl glycerol | 3996 | 630 | - | - | 0.74 | - | - | - | - | trace | - | - | 0.10 |
| 2-Acetyl-3-*p*-coumaroyl-2-feruloyl glycerol* | >4000 | 600 | - | - | 3.13 | - | - | - | - | 1.36 | 0.49 | - | - |
| 1-Acetyl-3-*p*-coumaroyl-2-caffeoyl glycerol* | >4000 | 600 | - | - | 4.84 | - | - | - | - | 0.94 | trace | - | - |
| 1,3-Diferoyl glycerol* | >4000 |  | - | - | 15.1 | - | - | - | - | 0.12 | 0.01 | - | - |
| 2-Acetyl-1,3-diferoyl glycerol* | >4000 | 630 | - | - | 5.46 | - | - | - | - | 0.42 | 0.44 | - | - |
| 2-Acetyl-1-*p*-coumaroyl-3-feruloyl glycerol* | >4000 | 630 | - | - | 2.25 | - | - | - | - | 0.23 | trace | 0.55 | - |
| 2-Acetyl-1-caffeoyl-3-*p*-coumaroyl glycerol* | >4000 | - | - | - | 3.7 | - | - | - | - | 0.40 | trace | 0.86 | - |
| 2-Acetyl-1,3-dicaffeoyl glycerol* | >4000 | - | - | - | 1.78 | - | - | - | - | - | - | - | - |
| Tetracosyl hexadecanoate | >4000 | 592 | - | - | 2.25 | - | 1.37 | - | - | 1.35 | 2.39 | 3.28 | 1.33 |
| Hexacosyl hexadecanoate | >4000 | 620 | - | - | - | - | - | - | - | - | - | 1.70 | 2.56 |

“Aspen-type” propolis Pr-1

“Aspen-birch-type” propolis Pr-3

“Birch-type” propolis Pr-5

“Poplar-type” propolis Pr-9
